# Toll‐like receptor 2 has a prominent but nonessential role in innate immunity to *Staphylococcus aureus* pneumonia

**DOI:** 10.14814/phy2.13491

**Published:** 2017-11-15

**Authors:** Shawn J. Skerrett, Marissa H. Braff, H. Denny Liggitt, Craig E. Rubens

**Affiliations:** ^1^ Department of Medicine University of Washington Seattle Washington; ^2^ Seattle Children's Hospital Research Institute Seattle Washington; ^3^ Department of Comparative Medicine University of Washington Seattle, Washington; ^4^ Present address: Gilead Sciences Seattle Washington 98102

**Keywords:** Alveolar macrophages, bacterial pneumonia, innate immunity, *Staphylococcus aureus*, toll‐like receptor 2

## Abstract

*Staphylococcus aureus* is an important cause of acute bacterial pneumonia. Toll‐like receptor 2 (TLR2) recognizes multiple components of the bacterial cell wall and activates innate immune responses to gram‐positive bacteria. We hypothesized that TLR2 would have an important role in pulmonary host defense against *S. aureus*. TLR null (TLR2^−/−^) mice and wild type (WT) C57BL/6 controls were challenged with aerosolized *S. aureus* at a range of inocula for kinetic studies of cytokine and antimicrobial peptide expression, lung inflammation, bacterial killing by alveolar macrophages, and bacterial clearance. Survival was measured after intranasal infection. Pulmonary induction of most pro‐inflammatory cytokines was significantly blunted in TLR2^−/−^ mice 4 and 24 h after infection in comparison with WT controls. Bronchoalveolar concentrations of cathelicidin‐related antimicrobial peptide also were reduced in TLR2^−/−^ mice. Lung inflammation, measured by enumeration of bronchoalveolar neutrophils and scoring of histological sections, was significantly blunted in TLR2^−/−^ mice. Phagocytosis of *S. aureus* by alveolar macrophages in vivo after low‐dose infection was unimpaired, but viability of ingested bacteria was significantly greater in TLR2^−/−^ mice. Bacterial clearance from the lungs was slightly impaired in TLR2^−/−^ mice after low‐dose infection only; bacterial elimination from the lungs was slightly accelerated in the TLR2^−/−^ mice after high‐dose infection. Survival after high‐dose intranasal challenge was 50–60% in both groups. TLR2 has a significant role in early innate immune responses to *S. aureus* in the lungs but is not required for bacterial clearance and survival from *S. aureus* pneumonia.

## Introduction

*Staphylococcus aureus* is a leading cause of health care associated pneumonia and is growing in importance as a respiratory pathogen in the community (Kollef et al. 2005; Chambers and Deleo 2009; Jones 2010). The worldwide emergence of highly virulent, antibiotic resistant‐strains of *S. aureus* lends special urgency to understanding mechanisms of host resistance to this vigorous pathogen (Zetola et al. 2005; DeLeo and Chambers 2009). Successful defense against *S. aureus* infections of the lungs and other tissues is largely dependent on an innate immune response that recruits neutrophils to the site of infection (Foster 2005; von Kockritz‐Blickwede et al. 2008; Robertson et al. 2008; Kohler et al. 2011).

The activation of innate immune responses to bacteria in the lungs involves the detection of bacterial ligands by pattern recognition sensors such as Toll‐like receptors (TLRs) (Fournier and Philpott 2005; Kawai and Akira 2011). Of the TLRs that have been identified, TLR2 has emerged as playing the most important role in the activation of innate immune responses to *S. aureus* (Fournier and Philpott 2005; Fournier 2012). TLR2 recognizes diverse microbial structures and detects multiple components of the staphylococcal cell wall, including lipoteichoic acid and lipopeptides (Lien et al. 1999; Schwandner et al. 1999; Takeuchi et al. 1999; Underhill et al. 1999; Yoshimura et al. 1999; Fournier and Philpott 2005; Hashimoto et al. 2006b; Kurokawa et al. 2009; Muller‐Anstett et al. 2010; Fournier 2012). TLR2 mediates the secretion of pro‐inflammatory cytokines and chemokines by macrophages stimulated with *S. aureus* (Underhill et al. 1999; Ozinsky et al. 2000; Hoebe et al. 2005; Schmaler et al. 2009; Ip et al. 2010; Wolf et al. 2011; Yimin et al. 2013), and facilitates the recruitment of neutrophils to diverse sites of *S. aureus* infection (Miller et al. 2006; Mullaly and Kubes 2006; Sun et al. 2006; Nichols et al. 2009). TLR2 also stimulates the killing of *S. aureus* by neutrophils (Jann et al. 2011; Yipp et al. 2012). Overall, the importance of TLR2 in host resistance to *S. aureus* appears to vary with the locus of infection. TLR2 has been shown to exert a protective effect against intravenous or subcutaneous challenge with *S. aureus* (Takeuchi et al. 2000; Hoebe et al. 2005; Miller et al. 2006; Gillrie et al. 2010; Yimin et al. 2013), but to be dispensable or counter‐protective in peritoneal infection or brain abscess (Kielian et al. 2005; Mullaly and Kubes 2006; Nichols et al. 2009; Blanchet et al. 2014). The role of TLR2 in host defense against *S. aureus* pneumonia has not been fully investigated.

Functional TLR2 is expressed by diverse cell populations within the lungs (Opitz et al. 2010), including alveolar macrophages (Hoogerwerf et al. 2010; Juarez et al. 2010; Kapetanovic et al. 2011), airway epithelial cells (Hertz et al. 2003; Armstrong et al. 2004; Muir et al. 2004; Sha et al. 2004; Soong et al. 2004; Mayer et al. 2007), and pulmonary endothelial cells (Pai et al. 2012). TLR2 has been shown to mediate pro‐inflammatory cytokine responses of alveolar macrophages and airway epithelial cells to *S. aureus* in vitro (Muir et al. 2004; Soong et al. 2004; Kapetanovic et al. 2011), as well as pulmonary inflammatory responses to staphylococcal lipoteichoic acid in vivo (Knapp et al. 2008). Thus, we hypothesized that TLR2 would play an important role in the activation of innate immune responses to *S. aureus* infection of the lungs. We used a murine model of acute *S. aureus* pneumonia to test this hypothesis (Skerrett et al. 2004; Ventura et al. 2008a,b).

## Materials and Methods

### Bacteria

A human blood isolate of *S. aureus* designated JP1 was obtained from the microbiology laboratory of the Veterans Affairs Puget Sound Health Care System (Skerrett et al. 2004; Ventura et al. 2008a,b). This is a serotype 8, methicillin‐sensitive strain that produces alpha toxin (Chaffin et al. 2012). The genetic features of this organism in comparison with other strains of *S. aureus*, its transcriptomic response to pulmonary infection, and other host: pathogen interactions during experimental pneumonia have been described (Skerrett et al. 2004; Braff et al. 2007; Ventura et al. 2008a,b; Chaffin et al. 2012). The organism was grown overnight in Luria Bertani (LB) broth, diluted to 30% glycerol, aliquoted, flash frozen in dry ice and ethanol, and stored at −80°C. For each experiment, bacteria were seeded in LB broth and incubated for 6 h at 37°C on a shaking platform, then diluted 100‐fold into fresh LB broth. After 16–18 h incubation at 37°C with agitation, stationary phase bacteria were pelleted by centrifugation, washed twice in PBS, and suspended in PBS to a concentration estimated by optical density at 540 nm and confirmed by quantitative culture on LB agar.

### Mice

Mice with targeted deletions of TLR2 were obtained from S. Akira (Osaka, Japan) and backcrossed to C57BL/6 mice for at least six generations, as described (Takeuchi et al. 2000; Skerrett et al. 2007). Male and female C57BL/6 mice 8–10 weeks of age and free of specific pathogens were purchased from Jackson Laboratories (Bar Harbor, ME) and served as wild type (WT) controls after being housed locally for at least 1 week. Mice were housed in laminar flow cages and permitted ad lib access to sterile food and water. All experiments were approved by the Institutional Animal Care and Use Committee of the University of Washington.

### Experimental pneumonia and tissue harvest

Two methods of infection were used. For kinetic studies of bacterial clearance and host responses, mice were exposed to aerosolized bacteria in a whole animal exposure chamber, as described (Skerrett et al. 2004). Briefly, groups of WT and TLR2^−/−^ mice were placed in individual wire mesh compartments within a 55L chamber and exposed for 30 min to bacterial aerosols generated by twin jet nebulizers (Salter, Arvin, CA). Three distinct inocula were tested (low, intermediate, and high), whereby the volume of broth in which the bacteria were cultured determined the concentration of bacteria in the final 20 mL volume of slurry used in the nebulizers. Actual bacterial deposition in each experiment was determined by quantitative culture of lung tissue harvested from sentinel mice immediately after infection (Table 1). At serial time points after infection (4 h, 24 h, 48 h, and 96 h), 3–5 mice in each group were euthanized with pentobarbital and exsanguinated by cardiac puncture. The left lung was harvested for quantitative culture and cytokine measurements, the spleen was taken for quantitative culture, and the right lung was lavaged by sequential instillation and retrieval of four 0.5 mL volumes of 0.9% sodium chloride with 0.6 mmol/L EDTA via a 22 g catheter in the trachea, as described (Morris et al. 2009). For survival studies mice were infected by the intranasal route, which permitted deposition of higher concentrations of bacteria in the lungs. Mice were anesthetized with isoflurane, held in the vertical position, and a 50 *μ*L suspension containing 3 × 10^8^ CFU bacteria in PBS was distributed between both nares (Ventura et al. 2008a,b). Mice were held upright for 1 min after inoculation and then were recovered in the prone position. Preliminary studies established that this dose of bacteria resulted in approximately 50% mortality in WT mice.

**Table 1:** Preparation and lung deposition of aerosolized *Staphylococcus aureus*

Inoculum	Volume of broth	Experiment	Nebulizer slurry (CFU/mL)	Lung deposition^a^ (CFU/lung)
Low	100 mL	1	8.8 × 10^10^	3.5 ± 0.1 × 10^5^
		2	7.5 × 10^10^	5.3 ± 1.4 × 10^5^
Intermediate	1 L	1	1.4 × 10^12^	6.6 ± 0.7 × 10^6^
		2	1.1 × 10^12^	2.9 ± 0.1 × 10^6^
High	2 L	1	3.2 × 10^12^	1.0 ± 0.03 × 10^7^
		2	3.2 × 10^12^	1.7 ± 0.2 × 10^7^

a Data are mean ± SEM, *n* = 3–4 mice, from which the left lung was homogenized and quantitatively cultured immediately after infection.

### Bacterial clearance

Left lungs and spleens each were homogenized in 1 mL PBS, and 0.1 mL volumes of serial dilutions in PBS were cultured on duplicate LB agar plates. Colonies were counted after 48 h incubation at 37°C.

### Bronchoalveolar lavage cell counts

At designated time points after infection mice underwent bronchoalveolar lavage (BAL) (Morris et al. 2009). The lavage fluid was centrifuged at 300 ***g*** and supernatants were stored at −80°C. The cell pellets were resuspended in RPMI 1640 (Mediatech, Manassas, VA) containing 10% heat‐inactivated fetal bovine serum (HyClone Laboratories, Logan, UT). Cell counts were measured by hemocytometer and differential counts were determined by examination of cytocentrifuge specimens stained with a modified Wright‐Giemsa technique (Diff‐Quick, Dade Behring, Dudingen, Switzerland).

### Measurement of cytokines, cathelicidin, and total protein

Lung homogenates in PBS were diluted 1:1 in lysis buffer containing 2× protease inhibitor cocktail (Roche Diagnostics, Mannheim, Germany), incubated on ice for 30 min, and then centrifuged at 1500 ***g***. The supernatants were harvested and stored at −80°C until assayed. Tumor necrosis factor‐*α* (TNF‐*α*), interleukin (IL)‐1*β*, IL‐6, IL‐10, IL‐12 p70, macrophage inflammatory protein 2 (MIP‐2; CXCL2), keratinocyte chemokine (KC; CXCL1), monocyte chemoattractant protein 1 (MCP‐1; CCL2), granulocyte‐macrophage colony stimulating factor (GM‐CSF), and interferon‐*γ* (IFN‐*γ*) were measured in lung homogenates by multiplex microbead array using a Luminex 100 analyzer (Austin, TX) and reagents purchased from R&D Systems (Minneapolis, MN). IL‐1*α* and IL‐17A were measured by ELISA using DuoSet kits from R&D Systems. Lung specimens from uninfected mice were used as controls. Mouse cathelicidin‐related antimicrobial peptide (CRAMP) was measured in BALF by ELISA, using reagents generously provided by R. Gallo (University of California, San Diego) (Braff et al. 2007). Total protein in BALF was measured using the bicinchoninic acid assay (Pierce, Rockford, IL).

### Histopathology

A separate experiment was performed to obtain specimens for histological analysis. Four and 24 h after exposure to aerosolized bacteria (intermediate dose), four WT and four TLR2^−/−^ mice were euthanized and exsanguinated. The trachea was cannulated, the chest opened, and the lungs were inflated to 20 cm pressure with 4% paraformaldehyde. The lungs were removed en bloc and fixed in 4% paraformaldehyde at 4°C for 24 h then transferred to 70% ethanol. The tissue was embedded in paraffin and stained with hematoxylin and eosin. Four widely spaced sections of lung from each animal were scored for the intensity of peribronchial, perivascular, and alveolar inflammation and necrosis, each on a scale of 0–4, by a veterinary pathologist who was blinded to the genotype and time after infection of each specimen (Morris et al. 2009; Gibson‐Corley et al. 2013).

### Phagocytosis and killing of *S. aureus* by alveolar macrophages in vivo

Mice were exposed to aerosolized *S. aureus* at the low inoculum, a concentration that resulted in pulmonary deposition of bacteria below the threshold required to elicit a rapid neutrophil response (Toews et al. 1979). Both lungs were lavaged 4 h after infection as described (Skerrett et al. 1999). The BALF from each mouse was centrifuged at 300 ***g*** at 4°C, and the supernatant stored at −80°C. The cell pellet was washed twice in cold Hank's Balanced Salt Solution and resuspended in 0.5 mL RPMI 1640. A 25 *μ*L volume of this suspension was removed for counting in a hemocytometer and 75 *μ*L were used for preparation of a cytocentrifuge slide stained with Diff‐Quik. The remaining cell suspension was lysed with 0.5% Triton X and quantitatively cultured. Preliminary studies established that this concentration of Triton X did not affect the viability of *S. aureus*. Complete lysis of the cell suspension was documented by microscopic analysis. The cytocentrifuge slides were scored for differential cell counts, the proportion of cells with cell‐associated bacteria, and the number of bacteria per cell (Skerrett et al. 2004).

### Statistical analysis

Statistical analyses were performed with GraphPad Prism 7 software (La Jolla, CA). Interval data are expressed as mean ± SEM, and statistical comparisons between two experimental groups were made using unpaired two‐tailed *T* tests. Cytokine analyses were adjusted for multiple comparisons using the Holm‐Sidak method (GraphPad). Ordinal data (histological grading) are shown as median with interquartile range, and statistical comparisons were made using Mann–Whitney *U* tests. A *P* value of <0.05 was considered significant.

## Results

### Cytokine responses after inhalation of *S. aureus* were blunted in TLR2^−/−^ mice

**Figure 1:**
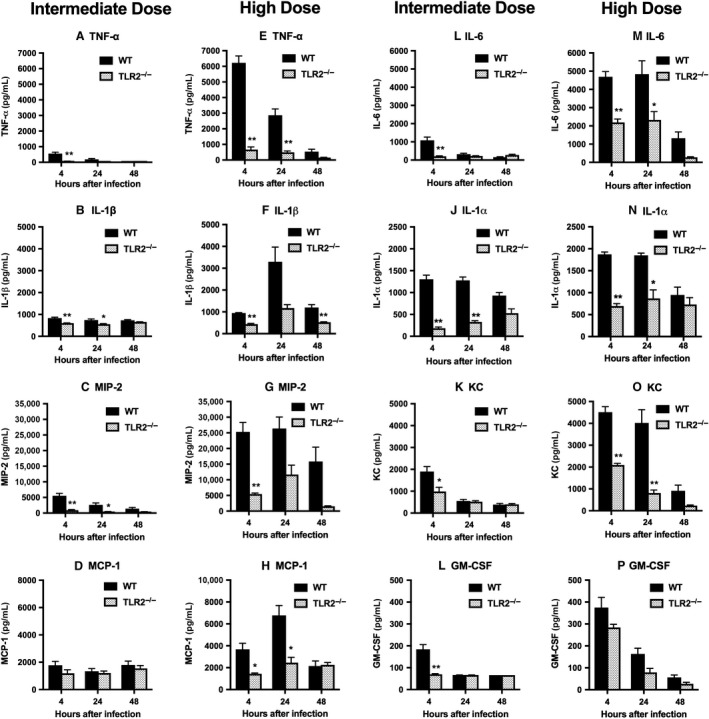
Lung cytokine responses to inhaled *Staphylococcus aureus* are blunted in TLR2^−/−^ mice. Cytokines were measured by microbead array or ELISA in lung homogenates harvested 4 h, 24 h, and 48 h after deposition of 4.5 × 10^6^ CFU/lung (intermediate dose; A–D, I–L) or 1.4 × 10^7^ CFU/lung (high dose; E–H, M–P). Data are mean ± SEM;* n* = 8 except for 48 h after intermediate dose and 4 h after high dose where *n* = 4; and represent the combined results of two separate experiments at each bacterial inoculum. *P < 0.05 versus WT; **P < 0.005 versus WT. Protein levels in uninfected lung homogenates were as follows (mean ± SEM,* n* = 4, all pg/mL): TNF‐*α*, 41 ± 9; IL‐1*β*, 311 ± 55; IL‐1*α*, 229 ± 27; MIP‐2 (CXCL2), 67 ± 43; KC (CXCL1), 264 ± 45; MCP‐1 (CCL2), 236 ± 97; IL‐6, 48 ± 7; GM‐CSF, 45 ± 13.

Levels of immunoreactive cytokines and chemokines were measured in lung homogenates harvested 4 h, 24 h, and 48 h after inhalation of *S. aureus* at two different inocula (Fig. 1). Dose‐ and time‐related induction of most proteins was evident in both WT and TLR2^−/−^ mice, but responses were significantly blunted in TLR2‐deficient animals. TNF‐*α*, MIP‐2, and IL‐1*α* concentrations at the 4 h and 24 h time points were reduced by >50% in TLR2^−/−^ mice at both levels of infectious challenge, suggesting that induction of these cytokines was largely dependent on TLR2. Lung levels of IL‐17A did not differ significantly between TLR2^−/−^ and WT mice, and concentrations of IL‐10, IL‐12p70, and IFN‐*γ* were not significantly above those in uninfected controls in either group of mice (not shown). These findings indicate that TLR2‐dependent recognition plays an important role in the activation of innate immune responses to *S. aureus* in the lungs.

### Lung inflammation in response to *S. aureus* infection was mildly reduced in TLR2^−/−^ mice

**Figure 2:**
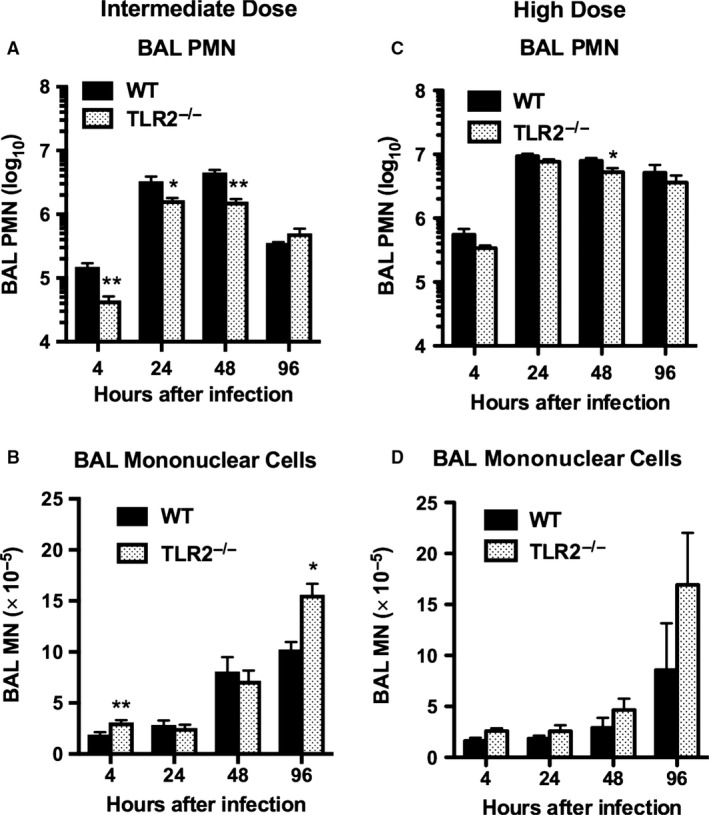
Reduced neutrophil response to inhaled *Staphylococcus aureus* in TLR2^−/−^ mice. Bronchoalveolar lavage (BAL) neutrophils (PMN) and mononuclear cells (MN) were determined at serial time points after inhalation of *S. aureus* at two different inocula: 4.5 × 10^6^ CFU/lung (intermediate dose; A, B), or 1.4 × 10^7^ CFU/lung (high dose; C, D). Data are mean ± SEM, *n* = 8, except for 48 h after intermediate dose and 4 h after high dose where *n* = 4; and represent the combined results of two separate experiments at each inoculum. *P < 0.05 versus WT; **P < 0.005 versus WT.

Inhalation of *S. aureus* resulted in a dose‐dependent influx of neutrophils into the airspaces of the lungs that was evident by 4 h after intermediate or high dose infection and persisted through 96 h of observation (Fig. 2; *P* < 0.005 intermediate versus high dose for both WT and TLR2^−/−^, at both 4 h and 24 h after infection). The number of bronchoalveolar neutrophils was reduced by more than 60% in TLR2^−/−^ mice in comparison with WT controls 4 h after intermediate dose infection and remained significantly blunted in this group through the first 48 h after infection (Fig. 2A). Greater numbers of mononuclear cells were present in BAL samples of TLR2^−/−^ mice 96 h after infection, suggesting delayed resolution of inflammation in these mice (Fig. 2A and B). After high dose challenge, in contrast, both WT and TLR2^−/−^ mice mounted vigorous neutrophilic responses; significantly fewer neutrophils were found in BAL samples from TLR2^−/−^ mice only at the 48 h time point, and there were no significant differences in mononuclear cells (Fig. 2C and D).

**Figure 3:**
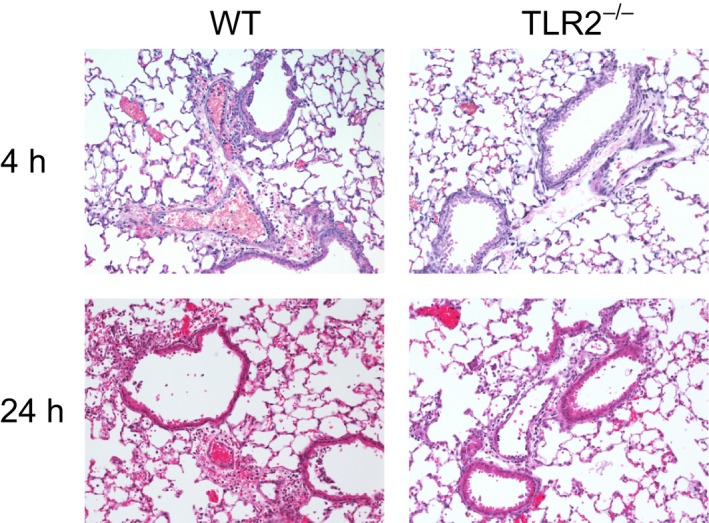
Reduced lung inflammation in TLR2^−/−^ mice with *Staphylococcus aureus* pneumonia. Lung tissue was harvested 4 h and 24 h after inhalation of *S. aureus* (6.6 × 10^6^ CFU/lung), sectioned, and stained with hematoxylin and eosin. Images of lesions representing the upper range of severity scores demonstrate less intense inflammation in TLR2^−/−^ mice.

**Figure 4:**
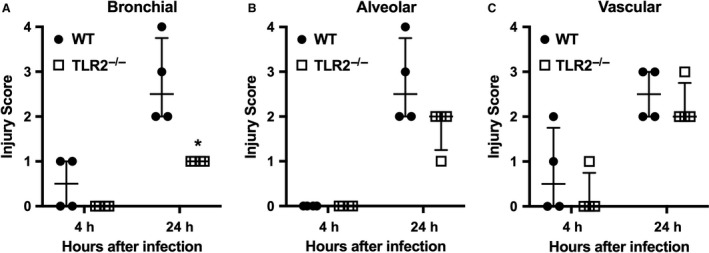
Reduced lung inflammation in TLR2^−/−^ mice with *Staphylococcus aureus* pneumonia. Lung tissue was harvested 4 h and 24 h after inhalation of *S. aureus* (6.6 × 10^6^ CFU/lung). Hematoxylin and eosin stained sections were scored for A. Bronchial, B. Alveolar, and C. Vascular inflammation and necrosis on a 4 point scale. Each data point represents the composite score from examination of 4 widely spaced sections of lung from an individual mouse. *P < 0.05 versus WT.

Reduced lung inflammation in TLR2^−/−^ mice also was evident from histological analysis. As shown in Figure 3, bronchiolar and alveolar inflammation was readily apparent 24 h after intermediate dose infection in WT mice, but these lesions were less severe in TLR2^−/−^ animals, in agreement with the BAL findings. Using semi‐quantitative morphometry, only bronchiolar inflammation 24 h after inhalation of *S. aureus* was significantly reduced in TLR2^−/−^ mice in comparison with WT mice (Fig. 4).

### Cathelicidin release into airways after inhalation of *S. aureus* was decreased in TLR2^−/−^ mice

**Figure 5:**
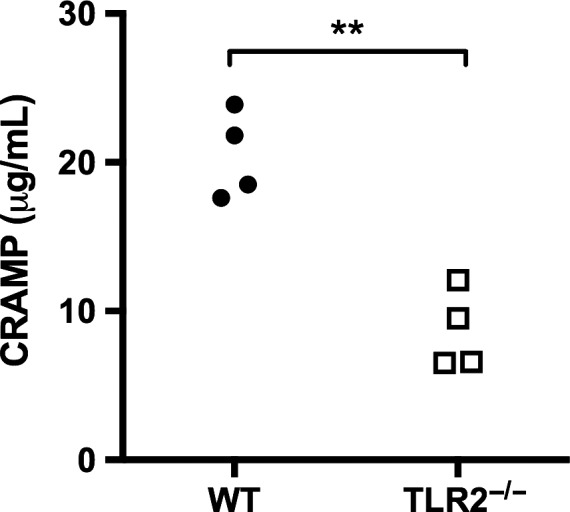
Decreased release of cathelicidin‐related antimicrobial peptide (CRAMP) in TLR2^−/−^ mice. CRAMP was measured by ELISA in bronchoalveolar lavage fluid 24 h after inhalation of *Staphylococcus aureus* (deposition 6.6 × 10^6^ CFU/lung). Each data point represents an individual mouse. **P = 0.001

We measured airway levels of CRAMP because this antimicrobial peptide has microbicidal activity against *S. aureus* (Bals et al. 1999; Jann et al. 2009; Alalwani et al. 2010), is inducible by TLR2 ligands (Liu et al. 2006), and is strongly upregulated in the lungs of mice with *S. aureus* pneumonia (Braff et al. 2007). Concentrations of CRAMP were undetectable in BALF from uninfected mice and in BALF harvested 4 h after infection with an intermediate dose of *S. aureus* (6.6 × 10^6^ CFU/lung). By 24 h after infection CRAMP was readily detectable in BALF from both WT and TLR2^−/−^ mice, but levels were reduced by more than 50% in TLR2^−/−^ mice in comparison with WT mice (Fig. 5). Thus, the release of CRAMP into the airways after inhalation of *S. aureus* is significantly impaired in the absence of TLR2.

### The viability of *S. aureus* ingested by alveolar macrophages in vivo was increased in TLR2^−/−^ mice

**Figure 6:**
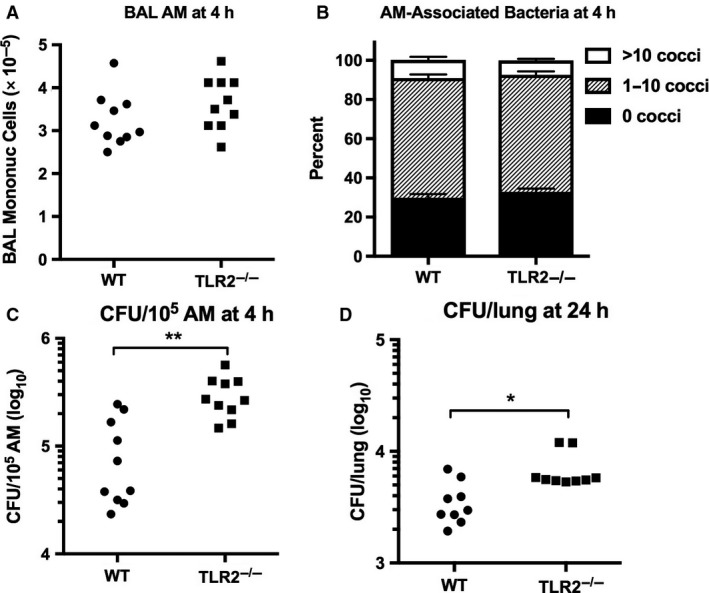
Increased viability of *Staphylococcus aureus* ingested by TLR2‐deficient alveolar macrophages (AM) in vivo. Bronchoalveolar cells (≥99% AM) were harvested 4 h after low‐dose airborne infection with *S. aureus* (deposition 4.3 × 10^5^ CFU/lung) (A). Cytocentrifuge samples were scored for the number of cell‐associated bacteria (B, mean ± SEM, *n* = 10). The cell pellets were lysed and quantitatively cultured (C). Lung homogenates were cultured 24 h after low‐dose infection (D). Data represent the combined results of two independent experiments. *P < 0.05; **P < 0.005.

To assess the uptake and killing of *S. aureus* by alveolar macrophages in vivo, we exposed mice to low‐dose aerosols that resulted in bacterial depositions that were below the threshold required to elicit a rapid neutrophil response (Toews et al. 1979). Mice were lavaged 4 h after infection, yielding bronchoalveolar cell populations that were ≥99% alveolar macrophages (by Wright‐Giemsa morphology) in all animals. The number of BAL macrophages did not differ between the two groups of mice (Fig. 6A). Cell‐associated bacteria were visible in approximately 70% of alveolar macrophages from both groups of animals; there were no differences between WT and TLR2^−/−^ mice in the proportion of cells harboring bacteria or in the number of bacteria per cell (Fig. 6B). However, the viability of *S. aureus* in cells harvested from TLR2^−/−^ mice was significantly greater than in cells from WT mice (Fig. 6C). Culture of lung homogenates 24 h after low dose infection demonstrated that the bacterial burden in TLR2^−/−^ mice was approximately twofold higher than that of WT controls (Fig. 6D). These data suggest that TLR2 is required for optimal bacterial killing by alveolar macrophages after inhalation of *S. aureus* in vivo, contributing to a mild impairment or delay in bacterial clearance after low‐dose infection.

### Bacterial clearance was not impaired in TLR2^−/−^ mice after intermediate or high‐dose infection

**Figure 7:**
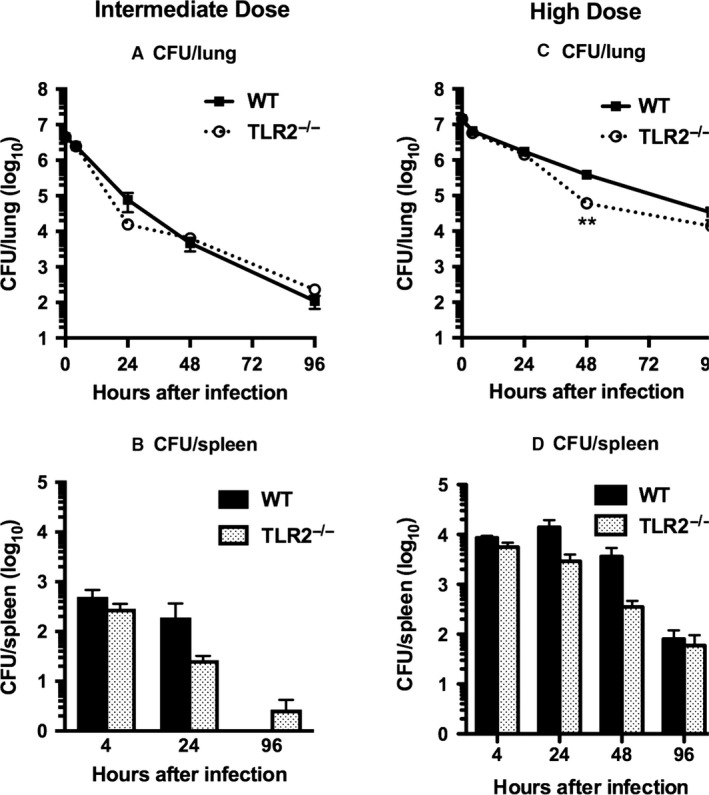
Bacterial clearance after inhalation of *Staphylococcus aureus* is unimpaired in TLR2^−/−^ mice after intermediate or high‐dose infection. Bacterial burdens in lung and spleen were measured at serial time points after deposition of 4.5 × 10^6^ CFU/lung (intermediate dose; A, B) or 1.4 × 10^7^ CFU/lung (high dose; C, D). Data are mean ± SEM, *n* = 8, except for 48 h after intermediate dose and 4 h after high dose where *n* = 4; and represent the combined results of two separate experiments at each inoculum. **P < 0.005 versus WT.

Despite the blunted inflammatory responses, reduced levels of CRAMP, and evidence of defective bacterial killing by alveolar macrophages, bacterial clearance from the lungs was not impaired in TLR2^−/−^ mice after inhalation of *S. aureus* at intermediate or high inocula (Fig. 7A and C). Indeed, bacterial clearance by TLR2^−/−^ mice was slightly more rapid after intermediate and high‐dose infection than in WT controls. By 96 h after infection, the number of bacteria persisting in the lungs was indistinguishable between the two groups of mice after both intermediate and high‐dose infection. Bacterial burdens in spleen, indicative of systemic spread of infection, did not differ significantly between the two groups of mice (Fig. 7B and D). Thus, TLR2 is not required for elimination of *S. aureus* from the lungs and spleen.

### Survival from staphylococcal pneumonia was not reduced in mice lacking TLR2

**Figure 8:**
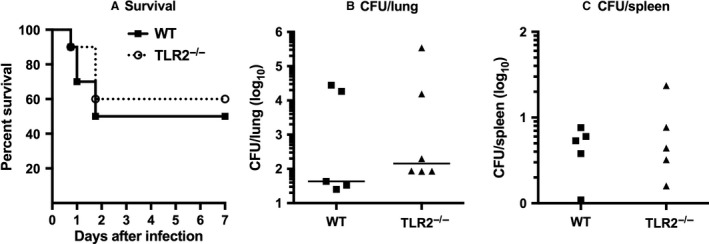
Survival from *Staphylococcus aureus* pneumonia is unimpaired in TLR2^−/−^ mice. Anesthetized mice (*n* = 10 per group) were inoculated intranasally with *S. aureus* (5 × 10^8^ CFU). After 7 days surviving mice were euthanized. Left lungs and spleens were homogenized for quantitative culture.

To determine if TLR2‐dependent signaling influenced the outcome of potentially lethal bolus infection, mice were infected by the intranasal route using an inoculum determined in pilot studies to result in approximately 50% survival in WT C57BL/6 mice. As shown in Figure 8, survival after infection did not differ significantly between TLR2^−/−^ and WT mice. Lung and spleen tissues harvested 7 days after challenge revealed persistent infection in all surviving mice, with no significant differences in bacterial burden between the two experimental groups.

## Discussion

Using a murine model of acute staphylococcal pneumonia, we found that the intrapulmonary production of proinflammatory cytokines and chemokines, the recruitment of neutrophils to the lungs, the release of cathelicidin into the airspaces, and bacterial containment by alveolar macrophages all were reduced in TLR2^−/−^ mice in comparison with WT controls. However, bacterial clearance and survival were not adversely affected by the absence of TLR2. These findings indicate that TLR2 has a prominent but non‐essential role in pulmonary host defense against *S. aureus* infection.

Our results demonstrate that TLR2 is involved in triggering innate immune responses to *S. aureus* in the lungs. TLR2 is known to recognize multiple ligands present in the cell walls of *S. aureus* (Lien et al. 1999; Schwandner et al. 1999; Yoshimura et al. 1999), including lipoproteins (Hashimoto et al. 2006a,b; Kurokawa et al. 2009), lipoteichoic acid (Schwandner et al. 1999; Travassos et al. 2004; Hashimoto et al. 2006a), and, possibly, peptidoglycan (Schwandner et al. 1999; Dziarski and Gupta 2005; Muller‐Anstett et al. 2010; Fournier 2012), as well as secreted toxins such as the Panton‐Valentine leukocidin (Zivkovic et al. 2011). Diverse resident cell populations in human and murine lungs express functional TLR2, including alveolar macrophages (Hoogerwerf et al. 2010; Juarez et al. 2010; Kapetanovic et al. 2011), airway epithelial cells (Hertz et al. 2003; Armstrong et al. 2004; Muir et al. 2004; Sha et al. 2004; Soong et al. 2004; Mayer et al. 2007), and pulmonary endothelial cells (Pai et al. 2012). In vitro studies have demonstrated that TLR2‐mediated recognition of intact *S. aureus* or its purified surface components stimulates NF*κ*B translocation and/or pro‐inflammatory cytokine production in alveolar macrophages and airway epithelial cells (Muir et al. 2004; Soong et al. 2004; Kapetanovic et al. 2011). In vivo experiments with mice have shown that intranasal instillation of staphylococcal lipoteichoic acid or peptidoglycan induces bronchoalveolar release of pro‐inflammatory cytokines and chemokines, associated with a rapid influx of neutrophils to the lungs (Leemans et al. 2002; Knapp et al. 2008; Poole et al. 2011). The response to LTA in these studies was demonstrated to be entirely dependent on TLR2 (Knapp et al. 2008). Similarly, endobronchial instillation of staphylococcal LTA in human volunteers stimulated local chemoattractant release and neutrophil recruitment (Hoogerwerf et al. 2008). Our data show that intrapulmonary production of pro‐inflammatory cytokines and chemokines in response to *S. aureus* infection was largely, albeit not completely, dependent on TLR2‐mediated signaling after both intermediate and high‐dose infection. These findings indicate that TLR2 plays an important but nonexclusive role in early recognition of *S. aureus* in the lungs.

The recruitment of neutrophils to the site of infection is an important early consequence of pathogen recognition, and neutrophils are required for effective resistance to *S. aureus* pneumonia (Robertson et al. 2008; Kohler et al. 2011; Robinson et al. 2014). TLR2 mediated signaling has been found to contribute to neutrophil recruitment in models of peritoneal, subcutaneous, corneal, and intracerebral infection with *S. aureus* (Miller et al. 2006; Mullaly and Kubes 2006; Sun et al. 2006; Nichols et al. 2009). Furthermore, intrapulmonary instillation of TLR2 ligands derived from *S. aureus* was found to induce an influx of neutrophils in both mice and humans (Leemans et al. 2002; Hoogerwerf et al. 2008; Knapp et al. 2008; Poole et al. 2011). TLR2‐mediated signaling also plays a role in the microbicidal actions of neutrophils. TLR2 contributes to the oxidative killing of *S. aureus* in vitro (Jann et al. 2011), and is required for neutrophil extracellular trap formation in response to *S. aureus* skin infection in vivo (Yipp et al. 2012). We found that neutrophilic inflammation in response to *S. aureus* lung infection was less dependent on TLR2 than the measured cytokine responses: early neutrophil recruitment was significantly blunted in TLR2^−/−^ mice after intermediate but not high‐dose infection. These results support a redundant and inoculum‐dependent function for TLR2 in the pulmonary inflammatory response to *S. aureus*.

We found that bronchoalveolar levels of cathelicidin‐related antimicrobial peptide (CRAMP) were significantly reduced in TLR2^−/−^ mice after inhalation of *S. aureus*. CRAMP is the murine homolog of the human cathelicidin, LL‐37 (Kovach et al. 2012; Vandamme et al. 2012; Beaumont et al. 2014). Cathelicidins are stored as inactive precursors in the secondary granules of neutrophils (Jann et al. 2009; Vandamme et al. 2012), and are inducible in other cell populations, including pulmonary macrophages and airway epithelial cells (Kovach et al. 2012; Vandamme et al. 2012). TLR2 ligands, in conjunction with vitamin D3, can stimulate cathelicidin expression in human monocytes and macrophages (Liu et al. 2006), and intraocular expression of CRAMP is blunted in TLR2^−/−^ mice with staphylococcal endophthalmitis (Talreja et al. 2015). Cathelicidins have broad microbicidal activities but also exert immunomodulatory effects that influence the recruitment and activation of neutrophils at sites of infection (Alalwani et al. 2010; Vandamme et al. 2012; Beaumont et al. 2014). These peptides exhibit direct antimicrobial activity against *S. aureus*, and contribute to killing of *S. aureus* by neutrophils (intracellularly) and airway epithelial cells (extracellularly) (Bals et al. 1999; Jann et al. 2009; Alalwani et al. 2010). Cathelicidins are expressed in the lungs after bacterial infection (Schaller‐Bals et al. 2002; Braff et al. 2007; Kovach et al. 2012), and CRAMP‐deficient mice exhibit impaired resistance to pulmonary infection with *Klebsiella pneumoniae* and *Pseudomonas aeruginosa* (Kovach et al. 2012; Beaumont et al. 2014). Chimera studies demonstrated that resistance to *K. pneumoniae* was dependent on expression of CRAMP by marrow derived rather than structural (parenchymal) cells (Kovach et al. 2012). We have previously reported that CRAMP accumulates in bronchoalveolar lavage fluid after infection with *S. aureus* (Braff et al. 2007), and in the present study demonstrate that this response is partially dependent on TLR2. However, our observations do not establish the source of cathelicidin in this model, nor define a specific role for cathelicidin in resistance to *S. aureus* pneumonia.

Alveolar macrophages can ingest and kill *S. aureus* and are largely responsible for the elimination of staphylococci from the lungs after low‐dose infection that falls below the threshold for triggering a neutrophil response (Green and Kass 1964; Goldstein et al. 1974; Toews et al. 1979; Onofrio et al. 1983; Jubrail et al. 2016). Our studies demonstrated that the avid phagocytosis of *S. aureus* by alveolar macrophages in vivo was unimpaired in the absence of TLR2. However, the viability of ingested bacteria 4 h after infection was significantly greater in alveolar macrophages from TLR2^−/−^ mice in comparison with WT controls, suggesting that the early killing of *S. aureus* by alveolar macrophages was partly dependent on TLR2. Prior in vitro studies with other macrophage populations have suggested a complex role for TLR2 in macrophage anti‐bacterial functions that may be cell‐specific. TLR2 was not required for the phagocytosis of *S. aureus* by bone marrow‐derived macrophages or peritoneal exudate macrophages, but differentially influenced the fate of intracellular bacteria in these cells (Blander and Medzhitov 2004; Watanabe et al. 2007). In bone marrow‐derived macrophages the fusion of *S. aureus*‐containing phagosomes with lysosomes was impaired in the absence of TLR2, indicating a TLR2‐dependent mechanism for phagosome maturation and bacterial disposal (Blander and Medzhitov 2004). On the other hand, superoxide release and bacterial killing after ingestion of *S. aureus* were enhanced in peritoneal exudate macrophages that lacked TLR2, supporting an inhibitory effect of TLR2 on the microbicidal activity of these cells (Watanabe et al. 2007).

Despite its role in triggering innate immune responses in the lungs, TLR2 was not required for resistance to *S. aureus* pneumonia. Survival from staphylococcal pneumonia was not impaired in mice lacking TLR2 and bacterial clearance from the lungs was minimally impaired in the absence of TLR2 only after low‐dose infection; indeed, elimination of *S. aureus* from the lungs was slightly accelerated in TLR2^−/−^ mice challenged with higher inocula of bacteria. Our results contrast with the only prior study of *S. aureus* pneumonia in TLR2^−/−^ mice, in which survival after intranasal infection was reduced in the absence of TLR2 (Blanchet et al. 2014). These data were presented in a supplemental figure restricted to survival analysis, which limits the context in which to draw comparisons with our observations (Blanchet et al. 2014). However, the investigators used a different strain of *S. aureus* and an inoculum more than threefold greater than the highest tested in our studies, suggesting that the contribution of TLR2 to the outcome of staphylococcal pneumonia may depend on the strain and infecting dose of bacteria. Defining the role of TLR2 in pulmonary defense against other isolates of *S. aureus* will require further investigation.

Research with different experimental models suggests that the role of TLR2 in resistance to *S. aureus* depends on the route of infection. After intravenous challenge, TLR2^−/−^ mice have consistently exhibited reduced survival (Takeuchi et al. 2000; Hoebe et al. 2005; Yimin et al. 2013), in association with increased burdens of bacteria in blood and kidneys, but not liver, spleen, or lungs (Takeuchi et al. 2000; Hoebe et al. 2005; Schmaler et al. 2009; Gillrie et al. 2010; Yimin et al. 2013). Furthermore, intravenous infection of mice with *S. aureus* expressing mutant lipoproteins that are unrecognized by TLR2 resulted in increased lethality and higher bacterial burdens in kidney and liver than infection with the wild‐type strain (Bubeck Wardenburg et al. 2006). Subcutaneous infection of TLR2^−/−^ mice with *S. aureus* resulted in increased local bacterial replication in comparison with wild‐type controls (Hoebe et al. 2005; Miller et al. 2006), without defective cytokine responses or neutrophil recruitment (Miller et al. 2006). Intra‐ocular infection with *S. aureus* led to blunted early cytokine responses, reduced neutrophil influx, and increased bacterial replication in TLR2^−/−^ mice compared with wild‐type controls (Talreja et al. 2015). In contrast, TLR2^−/−^ mice responded to intra‐peritoneal injection of *S. aureus* with a delayed inflammatory response but unimpaired bacterial clearance (Mullaly and Kubes 2006), and were protected from lethal challenge (Blanchet et al. 2014). In a murine model of staphylococcal brain abscess, the absence of TLR2 influenced cytokine responses and lymphocyte accumulation, but did not affect bacterial clearance, morbidity, or mortality (Kielian et al. 2005; Nichols et al. 2009). Collectively, these studies suggest that the role of TLR2 in mediating host resistance to *S. aureus* infection is tissue‐specific and inoculum‐dependent.

The contribution of TLR2 to pulmonary host defense against bacterial infection also appears to be pathogen‐specific. TLR2 partially mediates lung inflammatory responses to *Streptococcus pneumoniae*, but does not influence bacterial clearance or survival from experimental pneumococcal pneumonia (Knapp et al. 2004), similar to our findings with *S. aureus*. TLR2 does play an important role in host resistance to intracellular pathogens such as *Legionella pneumophila* and *Francisella tularensis* (Archer and Roy 2006; Hawn et al. 2006; Malik et al. 2006; Fuse et al. 2007; Abplanalp et al. 2009). In contrast, TLR2 has a modest suppressive effect on pulmonary defenses against extracellular gram‐negative pathogens such as *Pseudomonas aeruginosa* and *Acinetobacter baumanii* (Knapp et al. 2006; Skerrett et al. 2007).

TLR2 recognizes multiple surface components of *S. aureus* and plays a prominent role in the expression of innate immune responses to acute staphylococcal pneumonia. However, TLR2‐mediated signaling was not required for successful resistance to *S. aureus* in the lungs under the conditions tested. The contribution of TLR2 to host defense against *S. aureus* appears to depend on the site of infection.

## Conflict of Interest

None declared.

## References

[phy213491-bib-0001] Abplanalp, A. L. , I. R. Morris , B. K. Parida , J. M. Teale , and M. T. Berton . 2009 TLR‐dependent control of *Francisella tularensis* infection and host inflammatory responses. PLoS ONE 4:e7920. 19936231 10.1371/journal.pone.0007920 PMC2775407

[phy213491-bib-0002] Alalwani, S. M. , J. Sierigk , C. Herr , O. Pinkenburg , R. Gallo , C. Vogelmeier , et al. 2010 The antimicrobial peptide LL‐37 modulates the inflammatory and host defense response of human neutrophils. Eur. J. Immunol. 40:1118–1126. 20140902 10.1002/eji.200939275 PMC2908514

[phy213491-bib-0003] Archer, K. A. , and C. R. Roy . 2006 MyD88‐dependent responses involving toll‐like receptor 2 are important for protection and clearance of *Legionella pneumophila* in a mouse model of Legionnaires' disease. Infect. Immun. 74:3325–3333. 16714560 10.1128/IAI.02049-05 PMC1479235

[phy213491-bib-0004] Armstrong, L. , A. R. Medford , K. M. Uppington , J. Robertson , I. R. Witherden , T. D. Tetley , et al. 2004 Expression of functional toll‐like receptor‐2 and ‐4 on alveolar epithelial cells. Am. J. Respir. Cell Mol. Biol. 31:241–245. 15044215 10.1165/rcmb.2004-0078OC

[phy213491-bib-0005] Bals, R. , D. J. Weiner , R. L. Meegalla , and J. M. Wilson . 1999 Transfer of a cathelicidin peptide antibiotic gene restores bacterial killing in a cystic fibrosis xenograft model. J. Clin. Invest. 103:1113–1117. 10207162 10.1172/JCI6570 PMC408283

[phy213491-bib-0006] Beaumont, P. E. , B. McHugh , E. Gwyer Findlay , A. Mackellar , K. J. Mackenzie , R. L. Gallo , et al. 2014 Cathelicidin host defence peptide augments clearance of pulmonary *Pseudomonas aeruginosa* infection by its influence on neutrophil function in vivo. PLoS ONE 9:e99029. 24887410 10.1371/journal.pone.0099029 PMC4041793

[phy213491-bib-0007] Blanchet, C. , G. Jouvion , C. Fitting , J. M. Cavaillon , and M. Adib‐Conquy . 2014 Protective or deleterious role of scavenger receptors SR‐A and CD36 on host resistance to *Staphylococcus aureus* depends on the site of infection. PLoS ONE 9:e87927. 24498223 10.1371/journal.pone.0087927 PMC3909292

[phy213491-bib-0008] Blander, J. M. , and R. Medzhitov . 2004 Regulation of phagosome maturation by signals from toll‐like receptors. Science 304:1014–1018. 15143282 10.1126/science.1096158

[phy213491-bib-0009] Braff, M. H. , A. L. Jones , S. J. Skerrett , and C. E. Rubens . 2007 *Staphylococcus aureus* exploits cathelicidin antimicrobial peptides produced during early pneumonia to promote staphylokinase‐dependent fibrinolysis. J. Infect. Dis. 195:1365–1372. 17397009 10.1086/513277 PMC2366818

[phy213491-bib-0010] Bubeck Wardenburg, J. , W. A. Williams , and D. Missiakas . 2006 Host defenses against *Staphylococcus aureus* infection require recognition of bacterial lipoproteins. Proc. Natl Acad. Sci. USA 103:13831–13836. 16954184 10.1073/pnas.0603072103 PMC1564215

[phy213491-bib-0011] Chaffin, D. O. , D. Taylor , S. J. Skerrett , and C. E. Rubens . 2012 Changes in the *Staphylococcus aureus* transcriptome during early adaptation to the lung. PLoS ONE 7:e41329. 22876285 10.1371/journal.pone.0041329 PMC3410880

[phy213491-bib-0012] Chambers, H. F. , and F. R. Deleo . 2009 Waves of resistance: *staphylococcus aureus* in the antibiotic era. Nat. Rev. Microbiol. 7:629–641. 19680247 10.1038/nrmicro2200 PMC2871281

[phy213491-bib-0013] DeLeo, F. R. , and H. F. Chambers . 2009 Reemergence of antibiotic‐resistant *Staphylococcus aureus* in the genomics era. J. Clin. Invest. 119:2464–2474. 19729844 10.1172/JCI38226 PMC2735934

[phy213491-bib-0014] Dziarski, R. , and D. Gupta . 2005 *Staphylococcus aureus* peptidoglycan is a toll‐like receptor 2 activator: A reevaluation. Infect. Immun. 73:5212–5216. 16041042 10.1128/IAI.73.8.5212-5216.2005 PMC1201261

[phy213491-bib-0015] Foster, T. J. 2005 Immune evasion by staphylococci. Nat. Rev. Microbiol. 3:948–958. 16322743 10.1038/nrmicro1289

[phy213491-bib-0016] Fournier, B. 2012 The function of TLR2 during staphylococcal diseases. Front Cell. Infect. Microbiol. 2:167. 23316483 10.3389/fcimb.2012.00167 PMC3539681

[phy213491-bib-0017] Fournier, B. , and D. J. Philpott . 2005 Recognition of *Staphylococcus aureus* by the innate immune system. Clin. Microbiol. Rev. 18:521–540. 16020688 10.1128/CMR.18.3.521-540.2005 PMC1195972

[phy213491-bib-0018] Fuse, E. T. , K. Tateda , Y. Kikuchi , T. Matsumoto , F. Gondaira , A. Azuma , et al. 2007 Role of Toll‐like receptor 2 in recognition of *Legionella pneumophila* in a murine pneumonia model. J. Med. Microbiol. 56:305–312. 17314358 10.1099/jmm.0.46913-0

[phy213491-bib-0019] Gibson‐Corley, K. N. , A. K. Olivier , and D. K. Meyerholz . 2013 Principles for valid histopathologic scoring in research. Vet. Pathol. 50:1007–1015. 23558974 10.1177/0300985813485099 PMC3795863

[phy213491-bib-0020] Gillrie, M. R. , L. Zbytnuik , E. McAvoy , R. Kapadia , K. Lee , C. C. Waterhouse , et al. 2010 Divergent roles of Toll‐like receptor 2 in response to lipoteichoic acid and *Staphylococcus aureus* in vivo. Eur. J. Immunol. 40:1639–1650. 20306471 10.1002/eji.200939929

[phy213491-bib-0021] Goldstein, E. , W. Lippert , and D. Warshauer . 1974 Pulmonary alveolar macrophage. Defender against bacterial infection of the lung. J. Clin. Invest. 54:519–528. 4853956 10.1172/JCI107788 PMC301584

[phy213491-bib-0022] Green, G. M. , and E. H. Kass . 1964 The role of the alveolar macrophage in the clearance of bacteria from the lung. J. Exp. Med. 119:167–176. 14113111 10.1084/jem.119.1.167 PMC2137807

[phy213491-bib-0023] Hashimoto, M. , K. Tawaratsumida , H. Kariya , K. Aoyama , T. Tamura , and Y. Suda . 2006a Lipoprotein is a predominant Toll‐like receptor 2 ligand in *Staphylococcus aureus* cell wall components. Int. Immunol. 18:355–362. 16373361 10.1093/intimm/dxh374

[phy213491-bib-0024] Hashimoto, M. , K. Tawaratsumida , H. Kariya , A. Kiyohara , Y. Suda , F. Krikae , et al. 2006b Not lipoteichoic acid but lipoproteins appear to be the dominant immunobiologically active compounds in *Staphylococcus aureus* . J. Immunol. 177:3162–3169. 16920954 10.4049/jimmunol.177.5.3162

[phy213491-bib-0025] Hawn, T. R. , K. D. Smith , A. Aderem , and S. J. Skerrett . 2006 Myeloid differentiation primary response gene (88)‐ and toll‐like receptor 2‐deficient mice are susceptible to infection with aerosolized *Legionella pneumophila* . J. Infect. Dis. 193:1693–1702. 16703513 10.1086/504525

[phy213491-bib-0026] Hertz, C. J. , Q. Wu , E. M. Porter , Y. J. Zhang , K. H. Weismuller , P. J. Godowski , et al. 2003 Activation of Toll‐like receptor 2 on human tracheobronchial epithelial cells induces the antimicrobial peptide human beta defensin‐2. J. Immunol. 171:6820–6826. 14662888 10.4049/jimmunol.171.12.6820

[phy213491-bib-0027] Hoebe, K. , P. Georgel , S. Rutschmann , X. Du , S. Mudd , K. Crozat , et al. 2005 CD36 is a sensor of diacylglycerides. Nature 433:523–527. 15690042 10.1038/nature03253

[phy213491-bib-0028] Hoogerwerf, J. J. , A. F. de Vos , P. Bresser , J. S. van der Zee , J. M. Pater , A. de Boer , et al. 2008 Lung inflammation induced by lipoteichoic acid or lipopolysaccharide in humans. Am. J. Respir. Crit. Care Med. 178:34–41. 18403723 10.1164/rccm.200708-1261OC

[phy213491-bib-0029] Hoogerwerf, J. J. , A. F. de Vos , C. van't Veer , P. Bresser , A. de Boer , M. W. Tanck , et al. 2010 Priming of alveolar macrophages upon instillation of lipopolysaccharide in the human lung. Am. J. Respir. Cell Mol. Biol. 42:349–356. 19448156 10.1165/rcmb.2008-0362OC

[phy213491-bib-0030] Ip, W. K. , A. Sokolovska , G. M. Charriere , L. Boyer , S. Dejardin , M. P. Cappillino , et al. 2010 Phagocytosis and phagosome acidification are required for pathogen processing and MyD88‐dependent responses to *Staphylococcus aureus* . J. Immunol. 184:7071–7081. 20483752 10.4049/jimmunol.1000110 PMC2935932

[phy213491-bib-0031] Jann, N. J. , M. Schmaler , S. A. Kristian , K. A. Radek , R. L. Gallo , V. Nizet , et al. 2009 Neutrophil antimicrobial defense against *Staphylococcus aureus* is mediated by phagolysosomal but not extracellular trap‐associated cathelicidin. J. Leukoc. Biol. 86:1159–1169. 19638500 10.1189/jlb.0209053 PMC3192022

[phy213491-bib-0032] Jann, N. J. , M. Schmaler , F. Ferracin , and R. Landmann . 2011 TLR2 enhances NADPH oxidase activity and killing of *Staphylococcus aureus* by PMN. Immunol. Lett. 135:17–23. 20875459 10.1016/j.imlet.2010.09.007

[phy213491-bib-0033] Jones, R. N. 2010 Microbial etiologies of hospital‐acquired bacterial pneumonia and ventilator‐associated bacterial pneumonia. Clin. Infect. Dis. 51(Suppl 1):S81–S87. 20597676 10.1086/653053

[phy213491-bib-0034] Juarez, E. , C. Nunez , E. Sada , J. J. Ellner , S. K. Schwander , and M. Torres . 2010 Differential expression of Toll‐like receptors on human alveolar macrophages and autologous peripheral monocytes. Respir. Res. 11:2. 20051129 10.1186/1465-9921-11-2 PMC2817655

[phy213491-bib-0035] Jubrail, J. , P. Morris , M. A. Bewley , S. Stoneham , S. A. Johnston , S. J. Foster , et al. 2016 Inability to sustain intraphagolysosomal killing of *Staphylococcus aureus* predisposes to bacterial persistence in macrophages. Cell. Microbiol. 18:80–96. 26248337 10.1111/cmi.12485 PMC4778410

[phy213491-bib-0036] Kapetanovic, R. , M. Parlato , C. Fitting , V. Quesniaux , J. M. Cavaillon , and M. Adib‐Conquy . 2011 Mechanisms of TNF induction by heat‐killed *Staphylococcus aureus* differ upon the origin of mononuclear phagocytes. Am. J. Physiol. Cell Physiol. 300:C850–C859. 21209364 10.1152/ajpcell.00187.2010

[phy213491-bib-0037] Kawai, T. , and S. Akira . 2011 Toll‐like receptors and their crosstalk with other innate receptors in infection and immunity. Immunity 34:637–650. 21616434 10.1016/j.immuni.2011.05.006

[phy213491-bib-0038] Kielian, T. , A. Haney , P. M. Mayes , S. Garg , and N. Esen . 2005 Toll‐like receptor 2 modulates the proinflammatory milieu in *Staphylococcus aureus*‐induced brain abscess. Infect. Immun. 73:7428–7435. 16239543 10.1128/IAI.73.11.7428-7435.2005 PMC1273898

[phy213491-bib-0039] Knapp, S. , C. W. Wieland , C. van't veer , O. Takeuchi , S. Akira , S. Florquin , et al. 2004 Toll‐like receptor 2 plays a role in the early inflammatory response to murine pneumococcal pneumonia but does not contribute to antibacterial defense. J. Immunol. 172:3132–3138. 14978119 10.4049/jimmunol.172.5.3132

[phy213491-bib-0040] Knapp, S. , C. W. Wieland , S. Florquin , R. Pantophlet , L. Dijkshoorn , N. Tshimbalanga , et al. 2006 Differential roles of CD14 and toll‐like receptors 4 and 2 in murine Acinetobacter pneumonia. Am. J. Respir. Crit. Care Med. 173:122–129. 16210672 10.1164/rccm.200505-730OC

[phy213491-bib-0041] Knapp, S. , S. von Aulock , M. Leendertse , I. Haslinger , C. Draing , D. T. Golenbock , et al. 2008 Lipoteichoic acid‐induced lung inflammation depends on TLR2 and the concerted action of TLR4 and the platelet‐activating factor receptor. J. Immunol. 180:3478–3484. 18292574 10.4049/jimmunol.180.5.3478

[phy213491-bib-0042] von Kockritz‐Blickwede, M. , M. Rohde , S. Oehmcke , L. S. Miller , A. L. Cheung , H. Herwald , et al. 2008 Immunological mechanisms underlying the genetic predisposition to severe *Staphylococcus aureus* infection in the mouse model. Am. J. Pathol. 173:1657–1668. 18974303 10.2353/ajpath.2008.080337 PMC2626378

[phy213491-bib-0044] Kohler, J. , K. Breitbach , C. Renner , A. K. Heitsch , A. Bast , N. van Rooijen , et al. 2011 NADPH‐oxidase but not inducible nitric oxide synthase contributes to resistance in a murine *Staphylococcus aureus* Newman pneumonia model. Microbes Infect. 13:914–922. 21635963 10.1016/j.micinf.2011.05.004

[phy213491-bib-0045] Kollef, M. H. , A. Shorr , Y. P. Tabak , V. Gupta , L. Z. Liu , and R. S. Johannes . 2005 Epidemiology and outcomes of health‐care‐associated pneumonia: Results from a large US database of culture‐positive pneumonia. Chest 128:3854–3862. 16354854 10.1378/chest.128.6.3854

[phy213491-bib-0046] Kovach, M. A. , M. N. Ballinger , M. W. Newstead , X. Zeng , U. Bhan , F. S. Yu , et al. 2012 Cathelicidin‐related antimicrobial peptide is required for effective lung mucosal immunity in Gram‐negative bacterial pneumonia. J. Immunol. 189:304–311. 22634613 10.4049/jimmunol.1103196 PMC3566644

[phy213491-bib-0047] Kurokawa, K. , H. Lee , K. B. Roh , M. Asanuma , Y. S. Kim , H. Nakayama , et al. 2009 The triacylated ATP binding cluster transporter substrate‐binding lipoprotein of *Staphylococcus aureus* functions as a native ligand for toll‐like receptor 2. J. Biol. Chem. 284:8406–8411. 19139093 10.1074/jbc.M809618200 PMC2659198

[phy213491-bib-0048] Leemans, J. C. , M. J. Vervoordeldonk , S. Florquin , K. P. van Kessel , and T. van der Poll . 2002 Differential role of interleukin‐6 in lung inflammation induced by lipoteichoic acid and peptidoglycan from *Staphylococcus aureus* . Am. J. Respir. Crit. Care Med. 165:1445–1450. 12016110 10.1164/rccm.2106045

[phy213491-bib-0049] Lien, E. , T. J. Sellati , A. Yoshimura , T. H. Flo , G. Rawadi , R. W. Finberg , et al. 1999 Toll‐like receptor 2 functions as a pattern recognition receptor for diverse bacterial products. J. Biol. Chem. 274:33419–33425. 10559223 10.1074/jbc.274.47.33419

[phy213491-bib-0050] Liu, P. T. , S. Stenger , H. Li , L. Wenzel , B. H. Tan , S. R. Krutzik , et al. 2006 Toll‐like receptor triggering of a vitamin D‐mediated human antimicrobial response. Science 311:1770–1773. 16497887 10.1126/science.1123933

[phy213491-bib-0051] Malik, M. , C. S. Bakshi , B. Sahay , A. Shah , S. A. Lotz , and T. J. Sellati . 2006 Toll‐like receptor 2 is required for control of pulmonary infection with *Francisella tularensis* . Infect. Immun. 74:3657–3662. 16714598 10.1128/IAI.02030-05 PMC1479238

[phy213491-bib-0052] Mayer, A. K. , M. Muehmer , J. Mages , K. Gueinzius , C. Hess , K. Heeg , et al. 2007 Differential recognition of TLR‐dependent microbial ligands in human bronchial epithelial cells. J. Immunol. 178:3134–3142. 17312161 10.4049/jimmunol.178.5.3134

[phy213491-bib-0053] Miller, L. S. , R. M. O'Connell , M. A. Gutierrez , E. M. Pietras , A. Shahangian , C. E. Gross , et al. 2006 MyD88 mediates neutrophil recruitment initiated by IL‐1R but not TLR2 activation in immunity against *Staphylococcus aureus* . Immunity 24:79–91. 16413925 10.1016/j.immuni.2005.11.011

[phy213491-bib-0054] Morris, A. E. , H. D. Liggitt , T. R. Hawn , and S. J. Skerrett . 2009 Role of Toll‐like receptor 5 in the innate immune response to acute *P. aeruginosa* pneumonia. Am. J. Physiol. Lung Cell. Mol. Physiol. 297:L1112–L1119. 19801452 10.1152/ajplung.00155.2009 PMC2793188

[phy213491-bib-0055] Muir, A. , G. Soong , S. Sokol , B. Reddy , M. I. Gomez , A. Van Heeckeren , et al. 2004 Toll‐like receptors in normal and cystic fibrosis airway epithelial cells. Am. J. Respir. Cell Mol. Biol. 30:777–783. 14656745 10.1165/rcmb.2003-0329OC

[phy213491-bib-0056] Mullaly, S. C. , and P. Kubes . 2006 The role of TLR2 in vivo following challenge with *Staphylococcus aureus* and prototypic ligands. J. Immunol. 177:8154–8163. 17114491 10.4049/jimmunol.177.11.8154

[phy213491-bib-0057] Muller‐Anstett, M. A. , P. Muller , T. Albrecht , M. Nega , J. Wagener , Q. Gao , et al. 2010 Staphylococcal peptidoglycan co‐localizes with Nod2 and TLR2 and activates innate immune response via both receptors in primary murine keratinocytes. PLoS ONE 5:e13153. 20949035 10.1371/journal.pone.0013153 PMC2951902

[phy213491-bib-0058] Nichols, J. R. , A. L. Aldrich , M. M. Mariani , D. Vidlak , N. Esen , and T. Kielian . 2009 TLR2 deficiency leads to increased Th17 infiltrates in experimental brain abscesses. J. Immunol. 182:7119–7130. 19454709 10.4049/jimmunol.0802656 PMC2713313

[phy213491-bib-0059] Onofrio, J. M. , G. B. Toews , M. F. Lipscomb , and A. K. Pierce . 1983 Granulocyte‐alveolar‐macrophage interaction in the pulmonary clearance of *Staphylococcus aureus* . Am. Rev. Respir. Dis. 127:335–341. 6830054 10.1164/arrd.1983.127.3.335

[phy213491-bib-0060] Opitz, B. , V. van Laak , J. Eitel , and N. Suttorp . 2010 Innate immune recognition in infectious and noninfectious diseases of the lung. Am. J. Respir. Crit. Care Med. 181:1294–1309. 20167850 10.1164/rccm.200909-1427SO

[phy213491-bib-0061] Ozinsky, A. , D. M. Underhill , J. D. Fontenot , A. M. Hajjar , K. D. Smith , C. B. Wilson , et al. 2000 The repertoire for pattern recognition of pathogens by the innate immune system is defined by cooperation between toll‐like receptors. Proc. Natl Acad. Sci. USA 97:13766–13771. 11095740 10.1073/pnas.250476497 PMC17650

[phy213491-bib-0062] Pai, A. B. , H. Patel , A. J. Prokopienko , H. Alsaffar , N. Gertzberg , P. Neumann , et al. 2012 Lipoteichoic acid from *Staphylococcus aureus* induces lung endothelial cell barrier dysfunction: Role of reactive oxygen and nitrogen species. PLoS ONE 7:e49209. 23166614 10.1371/journal.pone.0049209 PMC3499573

[phy213491-bib-0063] Poole, J. A. , T. A. Wyatt , T. Kielian , P. Oldenburg , A. M. Gleason , A. Bauer , et al. 2011 Toll‐like receptor 2 regulates organic dust‐induced airway inflammation. Am. J. Respir. Cell Mol. Biol. 45:711–719. 21278324 10.1165/rcmb.2010-0427OC PMC3208620

[phy213491-bib-0064] Robertson, C. M. , E. E. Perrone , K. W. McConnell , W. M. Dunne , B. Boody , T. Brahmbhatt , et al. 2008 Neutrophil depletion causes a fatal defect in murine pulmonary *Staphylococcus aureus* clearance. J. Surg. Res. 150:278–285. 18621398 10.1016/j.jss.2008.02.009 PMC2605623

[phy213491-bib-0065] Robinson, K. M. , K. J. McHugh , S. Mandalapu , M. E. Clay , B. Lee , E. V. Scheller , et al. 2014 Influenza A virus exacerbates *Staphylococcus aureus* pneumonia in mice by attenuating antimicrobial peptide production. J. Infect. Dis. 209:865–875. 24072844 10.1093/infdis/jit527 PMC3935471

[phy213491-bib-0066] Schaller‐Bals, S. , A. Schulze , and R. Bals . 2002 Increased levels of antimicrobial peptides in tracheal aspirates of newborn infants during infection. Am. J. Respir. Crit. Care Med. 165:992–995. 11934727 10.1164/ajrccm.165.7.200110-020

[phy213491-bib-0067] Schmaler, M. , N. J. Jann , F. Ferracin , L. Z. Landolt , L. Biswas , F. Gotz , et al. 2009 Lipoproteins in *Staphylococcus aureus* mediate inflammation by TLR2 and iron‐dependent growth in vivo. J. Immunol. 182:7110–7118. 19454708 10.4049/jimmunol.0804292

[phy213491-bib-0068] Schwandner, R. , R. Dziarski , H. Wesche , M. Rothe , and C. J. Kirschning . 1999 Peptidoglycan‐ and lipoteichoic acid‐induced cell activation is mediated by toll‐like receptor 2. J. Biol. Chem. 274:17406–17409. 10364168 10.1074/jbc.274.25.17406

[phy213491-bib-0069] Sha, Q. , A. Q. Truong‐Tran , J. R. Plitt , L. A. Beck , and R. P. Schleimer . 2004 Activation of airway epithelial cells by toll‐like receptor agonists. Am. J. Respir. Cell Mol. Biol. 31:358–364. 15191912 10.1165/rcmb.2003-0388OC

[phy213491-bib-0070] Skerrett, S. J. , T. R. Martin , E. Y. Chi , J. J. Peschon , K. M. Mohler , and C. B. Wilson . 1999 Role of the type 1 TNF receptor in lung inflammation after inhalation of endotoxin or *Pseudomonas aeruginosa* . Am. J. Physiol. 276:L715–L727. 10330027 10.1152/ajplung.1999.276.5.L715

[phy213491-bib-0071] Skerrett, S. J. , H. D. Liggitt , A. M. Hajjar , and C. B. Wilson . 2004 Cutting edge: Myeloid differentiation factor 88 is essential for pulmonary host defense against *Pseudomonas aeruginosa* but not *Staphylococcus aureus* . J. Immunol. 172:3377–3381. 15004134 10.4049/jimmunol.172.6.3377

[phy213491-bib-0072] Skerrett, S. J. , C. B. Wilson , H. D. Liggitt , and A. M. Hajjar . 2007 Redundant Toll‐like receptor signaling in the pulmonary host response to *Pseudomonas aeruginosa* . Am. J. Physiol. Lung Cell. Mol. Physiol. 292:L312–L322. 16936244 10.1152/ajplung.00250.2006

[phy213491-bib-0073] Soong, G. , B. Reddy , S. Sokol , R. Adamo , and A. Prince . 2004 TLR2 is mobilized into an apical lipid raft receptor complex to signal infection in airway epithelial cells. J. Clin. Invest. 113:1482–1489. 15146246 10.1172/JCI20773 PMC406530

[phy213491-bib-0074] Sun, Y. , A. G. Hise , C. M. Kalsow , and E. Pearlman . 2006 *Staphylococcus aureus*‐induced corneal inflammation is dependent on Toll‐like receptor 2 and myeloid differentiation factor 88. Infect. Immun. 74:5325–5332. 16926427 10.1128/IAI.00645-06 PMC1594867

[phy213491-bib-0075] Takeuchi, O. , K. Hoshino , T. Kawai , H. Sanjo , H. Takada , T. Ogawa , et al. 1999 Differential roles of TLR2 and TLR4 in recognition of gram‐negative and gram‐positive bacterial cell wall components. Immunity 11:443–451. 10549626 10.1016/s1074-7613(00)80119-3

[phy213491-bib-0076] Takeuchi, O. , K. Hoshino , and S. Akira . 2000 Cutting edge: tLR2‐deficient and MyD88‐deficient mice are highly susceptible to *Staphylococcus aureus* infection. J. Immunol. 165:5392–5396. 11067888 10.4049/jimmunol.165.10.5392

[phy213491-bib-0077] Talreja, D. , P. K. Singh , and A. Kumar . 2015 In vivo role of TLR2 and MyD88 signaling in eliciting innate immune responses in staphylococcal endophthalmitis. Invest. Ophthalmol. Vis. Sci. 56:1719–1732. 25678692 10.1167/iovs.14-16087 PMC4356198

[phy213491-bib-0078] Toews, G. B. , G. N. Gross , and A. K. Pierce . 1979 The relationship of inoculum size to lung bacterial clearance and phagocytic cell response in mice. Am. Rev. Respir. Dis. 120:559–566. 114074 10.1164/arrd.1979.120.3.559

[phy213491-bib-0079] Travassos, L. H. , S. E. Girardin , D. J. Philpott , D. Blanot , M. A. Nahori , C. Werts , et al. 2004 Toll‐like receptor 2‐dependent bacterial sensing does not occur via peptidoglycan recognition. EMBO Rep. 5:1000–1006. 15359270 10.1038/sj.embor.7400248 PMC1299148

[phy213491-bib-0080] Underhill, D. M. , A. Ozinsky , A. M. Hajjar , A. Stevens , C. B. Wilson , M. Bassetti , et al. 1999 The Toll‐like receptor 2 is recruited to macrophage phagosomes and discriminates between pathogens. Nature 401:811–815. 10548109 10.1038/44605

[phy213491-bib-0081] Vandamme, D. , B. Landuyt , W. Luyten , and L. Schoofs . 2012 A comprehensive summary of LL‐37, the factotum human cathelicidin peptide. Cell. Immunol. 280:22–35. 23246832 10.1016/j.cellimm.2012.11.009

[phy213491-bib-0082] Ventura, C. L. , R. Higdon , L. Hohmann , D. Martin , E. Kolker , H. D. Liggitt , et al. 2008a *Staphylococcus aureus* elicits marked alterations in the airway proteome during early pneumonia. Infect. Immun. 76:5862–5872. 18852243 10.1128/IAI.00865-08 PMC2583584

[phy213491-bib-0083] Ventura, C. L. , R. Higdon , E. Kolker , S. J. Skerrett , and C. E. Rubens . 2008b Host airway proteins interact with *Staphylococcus aureus* during early pneumonia. Infect. Immun. 76:888–898. 18195024 10.1128/IAI.01301-07 PMC2258841

[phy213491-bib-0084] Watanabe, I. , M. Ichiki , A. Shiratsuchi , and Y. Nakanishi . 2007 TLR2‐mediated survival of *Staphylococcus aureus* in macrophages: A novel bacterial strategy against host innate immunity. J. Immunol. 178:4917–4925. 17404273 10.4049/jimmunol.178.8.4917

[phy213491-bib-0085] Wolf, A. J. , A. Arruda , C. N. Reyes , A. T. Kaplan , T. Shimada , K. Shimada , et al. 2011 Phagosomal degradation increases TLR access to bacterial ligands and enhances macrophage sensitivity to bacteria. J. Immunol. 187:6002–6010. 22031762 10.4049/jimmunol.1100232 PMC3221871

[phy213491-bib-0043] Yimin , M. Kohanawa , S. Zhao , M. Ozaki , S. Haga , G. Nan , et al. 2013 Contribution of toll‐like receptor 2 to the innate response against *Staphylococcus aureus* infection in mice. PLoS ONE 8:e74287. 24058538 10.1371/journal.pone.0074287 PMC3772844

[phy213491-bib-0086] Yipp, B. G. , B. Petri , D. Salina , C. N. Jenne , B. N. Scott , L. D. Zbytnuik , et al. 2012 Infection‐induced NETosis is a dynamic process involving neutrophil multitasking in vivo. Nat. Med. 18:1386–1393. 22922410 10.1038/nm.2847 PMC4529131

[phy213491-bib-0087] Yoshimura, A. , E. Lien , R. R. Ingalls , E. Tuomanen , R. Dziarski , and D. Golenbock . 1999 Cutting edge: Recognition of Gram‐positive bacterial cell wall components by the innate immune system occurs via Toll‐like receptor 2. J. Immunol. 163:1–5. 10384090

[phy213491-bib-0088] Zetola, N. , J. S. Francis , E. L. Nuermberger , and W. R. Bishai . 2005 Community‐acquired meticillin‐resistant *Staphylococcus aureus*: an emerging threat. Lancet Infect. Dis. 5:275–286. 15854883 10.1016/S1473-3099(05)70112-2

[phy213491-bib-0089] Zivkovic, A. , O. Sharif , K. Stich , B. Doninger , M. Biaggio , J. Colinge , et al. 2011 TLR 2 and CD14 mediate innate immunity and lung inflammation to staphylococcal Panton‐Valentine leukocidin in vivo. J. Immunol. 186:1608–1617. 21178007 10.4049/jimmunol.1001665

